# Assessment of the reliability and quality of breast cancer related videos on TikTok and Bilibili: cross-sectional study in China

**DOI:** 10.3389/fpubh.2023.1296386

**Published:** 2024-01-22

**Authors:** Hui Liu, Jialun Peng, Lu Li, Ao Deng, Xiangxin Huang, Guobin Yin, Jia Ming, Haojun Luo, Yinyin Liang

**Affiliations:** ^1^Department of Breast and Thyroid Surgery, The Second Affiliated Hospital of Chongqing Medical University, Chongqing, China; ^2^Department of Hepatobiliary Surgery, The Second Affiliated Hospital of Chongqing Medical University, Chongqing, China

**Keywords:** breast cancer, video, cross-sectional study, health education, quality evaluation

## Abstract

**Background:**

As the most common malignant tumor in the world, breast cancer also brings a huge disease burden to China. Ordinary people are increasingly inclined to use the Internet, especially video social platforms, as a source of health information. Educating the public to obtain correct information is important to reduce the incidence of breast cancer and improve the prognosis. However, the quality and reliability of breast cancer-related video content have not been fully studied.

**Objective:**

This study aims to evaluate the quality of the information of breast cancer-related videos on TikTok and Bilibili video sharing platforms and factors related to video quality.

**Methods:**

We collected the top 100 videos about breast cancer on TikTok and Bilibili, respectively. Categorize videos according to video source and video content. Video quality and reliability were assessed using Global Quality Score (GQS) and modified DISCERN (mDISCERN) tools. We also analyzed the correlation between video quality and video likes, comments, saves, and shares.

**Results:**

Although the quality and reliability of Bilibili’s breast cancer videos were higher than TikTok (*p* = 0.002 and *p* = 0.001, respectively), the video quality of both video sharing platforms was not satisfactory, with a median GQS scores of 2.00 and 3.00 and mDISCERN scores of 1.00 and 2.00, respectively. In general, the quality and reliability of videos released by medical practitioners were higher than those of non-medical practitioners, and the quality and reliability of videos covering disease-related knowledge were higher than those of news reports (all *p* < 0.001). Among medical practitioners, the quality of videos uploaded by doctors in breast disease was significantly lower than that of doctors in other areas (*p* < 0.05). There was a significant positive correlation between video quality and duration (*r* = 0.240, *p* < 0.001), a weak negative correlation between video quality and likes (*r* = 0.191, *p* < 0.01), video quality and comments (*r* = 0.256, *p* < 0.001), video reliability and likes (*r* = 0.198, *p* < 0.001), video reliability and comments (*r* = 0.243, *p* < 0.01).

**Conclusion:**

Our study shows that the quality and reliability of breast cancer-related videos on TikTok and Bilibili are poor, and the overall quality is unsatisfactory. But videos uploaded by medical practitioners covering disease knowledge, prevention and treatment are of higher quality. Medical practitioners are encouraged to publish more high-quality videos, while video social platforms should formulate relevant policies to censor and supervise health education videos, so as to enable the public to obtain reliable health information.

## 1 Introduction

Breast cancer has now overtaken lung cancer as the most common malignant tumor in the world, new cases account for about 1/8 of all cancer cases, and about 1/6 of female cancer patients die of breast cancer (1). The incidence of breast cancer is increasing in China. In 2020, 416,371 Chinese women were diagnosed with breast cancer, and 117,174 Chinese women died of breast cancer, which put considerable pressure on China’s financial and medical systems (2). Adopting lifestyle changes such as reducing overweight, alcohol consumption and processed meat intake, and encouraging physical activity and breastfeeding, can reduce the risk of breast cancer (1, 3). Early detection, diagnosis and effective treatment of breast cancer are crucial to improving patient prognosis and reducing mortality. Therefore, it is essential to provide accurate and reliable information about breast cancer to guide the public.

With the development of Internet technology, the number of global Internet users has exceeded 5.4 billion by 2022, of which Chinese users account for 18.47% (4). An observable shift has occurred in the way people obtain medical information. Compared with traditional sources such as books and television, more and more people tend to look for disease-related information on the Internet (5–7). Previous studies have shown that 34–49% of breast cancer patients obtain cancer-related information on the Internet (8, 9). In recent years, information in the form of video has become more popular. According to statistics, videos with #cancer hashtags on TikTok have amassed over 1.1 billion views worldwide (10). Compared with traditional text messages, video social media can not only explain information more simply, but also stimulate users’ healthy behavior because of its rich visual effects (11, 12). Due to the abundance of content creators on video social platforms and video content is not subject to any monitoring or censorship process, the reliability of medical information provided based on videos has been questioned (13). Patients may inadvertently encounter incomplete or misleading information, potentially leading to erroneous medical decisions. Therefore, the censorship of online health videos is very necessary.

In China, TikTok, known as Douyin, is the video social media application with the largest number of users, covering food, travel, education, and other types of videos, boasting over 750 million daily active users (14). As a comprehensive video sharing platform highly popular among China’s young generation, Bilibili provides users with a large number of high-quality video resources, with 315 million monthly active users (15). The two platforms are currently the main medium for disseminating health information online in China, Breast cancer-related videos have been viewed 240 million times in TikTok and 60 million times in Bilibili. Previous researches have examined the quality of videos on different themes on TikTok and Bilibili, and there are differences in video quality among different themes. The quality of videos about liver cancer and inflammatory bowel disease is generally unsatisfactory (16, 17), but the quality and reliability of videos about plastic surgery are deemed satisfactory (18). Although breast cancer videos on YouTube and TikTok have been evaluated, we found a large number of videos about breast cancer on TikTok Chinese version and Bilibili, and the information presented has not been evaluated. To address this research gap, we evaluated and compared the content, quality, and reliability of breast cancer-related videos on TikTok and Bilibili.

## 2 Methods

### 2.1 Search strategy and data collection

**Figure 1:**
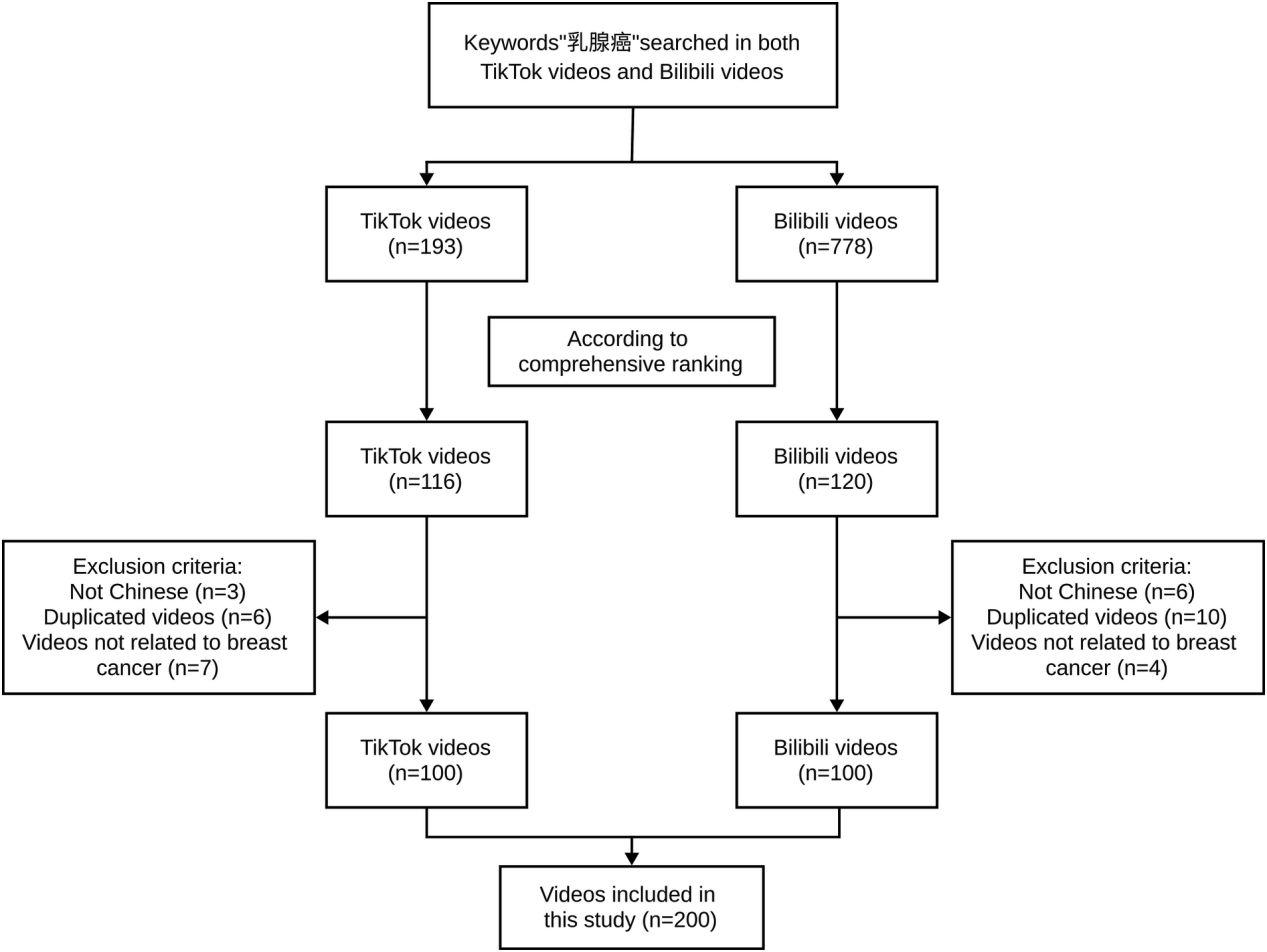
Search strategy for videos on breast cancer.

In this cross-sectional study, we used the keyword “乳腺癌” (“breast cancer” in Chinese) to search on TikTok (Chinese version 2.8.0) and Bilibili (Chinses version 1.11.2) on July 28, 2023 (Figure 1). In order to avoid bias caused by personalized recommendations, both platforms use newly registered accounts to conduct searches. We did not apply any filtering conditions to restrict the search. The videos were comprehensively sorted according to the TikiTok and Bilibili algorithms, after excluding non-Chinese videos, repetitive videos (the same content but different sources), and videos unrelated to breast cancer, we selected the top 100 videos.

In order to reduce the deviation caused by video updates over time, we extracted the basic information of each video on July 28, 2023, including video source, video content, duration (in seconds), upload date, and other viewer interaction quality markers including views, likes, comments, and shares. For accounts with professional logos, we not only carefully checked the account registration information of the video uploader, but also visited the corresponding hospital official website to ensure authenticity. The extracted data were recorded in Excel (Microsoft Inc).

### 2.2 Classification of videos

We divided the videos into 2 groups according to their source: (1) medical practitioners and (2) non-medical practitioners. Medical practitioners were further classified: (1) doctors in breast disease and (2) doctors in other disease fields; non-medical practitioners were further classified: (1) science communicators, (2) patients. According to the video content, we divide the videos into 5 groups: (1) disease knowledge, (2) treatment, (3) prevention, (4) news and reports and (5) advertisement and others. Specific classification details are showed in Table 1.

**Table 1:** Classification of videos.

Video source	
doctors in breast disease	Including doctors in breast surgery and internal breast medicine, surgical oncology and internal medical oncology, and other doctors who work on breast cancer related issues
doctors in other disease areas	Including other doctors in surgery, internal medicine, emergency medicine, interventional medicine, imaging and other areas of modern medicine
Science communicators	Including individual science communicators, non-profit organizations/institutions
Patients	Including breast cancer patients, family members of pancreatic cancer patients

Video content	
Disease knowledge	Including anatomical, pathologic, epidemiologic, and basic research related to breast cancer
Treatment	Any knowledge related to breast cancer treatment
Prevention	Preventative measures for breast cancer, including dietary prevention, risk factor control and other prevention-related knowledge
news report	Breast cancer related news, patient reports and patient clinic videos
Advertisement and others	Including commercials and other videos that do not reflect knowledge about breast cancer

### 2.3 Video assessment

We used modified DISCERN (mDISCERN) and Global Quality Score (GQS) tools to evaluate video reliability and information quality respectively, and their effectiveness has been confirmed in previous studies (19–21). mDISCERN tool was adapted from the DISCERN tool by Singh et al. to assess the reliability of video through five aspects (clarity, relevance, impartiality, stability, and plasticity), which shows better convenience and accuracy in evaluating video materials compared to the DISCERN tool (22, 23). The mDISCERN tool consists of five problems and gets a point for each problem solved, with a total score ranges from 0 to 5. The higher the score, the better the reliability. GQS was proposed by Bernard et al., originally used to evaluate the information quality of websites, and has now been widely used to evaluate the quality of video information (20, 24). GQS systematically evaluates the quality of video based on information quality, flow and usefulness, with scores ranging from 1 (very poor) to 5 (excellent) (24). The detailed scoring standards of GQS and mDISCERN are shown in Supplementary Tables S1–S3.

The video link was provided in a table format to two raters (JM and GY), who are two professional doctors who have been engaged in breast surgery for a long time. To reduce scoring error, the order of video links was disordered. Before evaluating the videos, two raters carefully read the scoring details of GQS and mDISCERN. Two raters independently viewed the videos at the same time to rate them and categorized the videos based on source and content. If the scores between the two raters were inconsistent, a full discussion was held with another observer (HL) to reach a consensus.

### 2.4 Statistical analyzes

The Shapiro Wilk test was used to test the normality of the data. Data with non-normal distribution were described statistically by median (IQR), and counting data were expressed by frequency and percentage. The Kruskal Wallis test evaluated differences between three or more groups of variables, and Dunn’s test was used for two-way between-group comparisons of variables that were not normally distributed. We used Cohen κ to evaluate the consistency between the two raters in GQS scores and mDISCERN scores, and Cohen *κ* ≥ 0.75 was considered to be consistent. A *p* value <0.05 was deemed statistically significant. We used Spearman correlation analysis to assess the relationship between non-normal variables. All statistical analyzes were performed by R software (R version 4.0.3).

## 3 Results

### 3.1 Basic characteristics of videos

After applying the inclusion and exclusion criteria, we reviewed and performed data statistics on 200 videos, 100 from TikTok and 100 from Bilibili. Table 2 is the basic feature of the videos. TikTok videos have more likes, comments, saves, shares and days since release than Bilibili videos (all *p* < 0.001), while Bilibili videos are longer than TikTok videos (*p* < 0.001).

**Table 2:** Baseline characteristics of the videos.

Characteristic	Release platform			*p*-value^b^
	Overall, *N* = 2,001^a^	TikTok, *N* = 1,001	Bilibili, *N* = 1,001	
GQS				0.002
Median (IQR)	2.0 (2.00, 3.00)	2.0 (2.00, 3.00)	3.0 (2.00, 3.00)	
mDISCERN				0.001
Median (IQR)	2.0 (1.00, 2.00)	1.0 (1.00, 2.00)	2.0 (1.00, 2.25)	
Likes				<0.001
Median (IQR)	3,430.0 (97.00, 29,750.00)	28,000.0 (7,830.00, 93,250.00)	95.0 (10.00, 503.75)	
Comments				<0.001
Median (IQR)	254.5 (8.50, 1,802.25)	1,626.5 (676.00, 5,364.25)	8.0 (1.00, 44.50)	
Saves				<0.001
Median (IQR)	245.0 (20.75, 1,458.75)	1,193.0 (347.00, 3,250.50)	24.0 (5.00, 135.50)	
Shares				<0.001
Median (IQR)	267.0 (8.75, 2,211.25)	2,185.0 (654.25, 7,119.50)	8.5 (1.00, 54.75)	
Days since published				<0.001
Median (IQR)	470.0 (240.00, 667.25)	553.0 (422.75, 699.00)	334.5 (155.00, 641.75)	
Duration				<0.001
Median (IQR)	97.5 (52.00, 197.25)	71.5 (48.00, 118.25)	158.5 (56.75, 314.50)	

a *n* (%).

b Wilcoxon rank sum test; Pearson’s Chi-squared test; Fisher’s exact test.

### 3.2 Main results

#### 3.2.1 Overall scoring and comparison of platform videos

**Figure 2:**
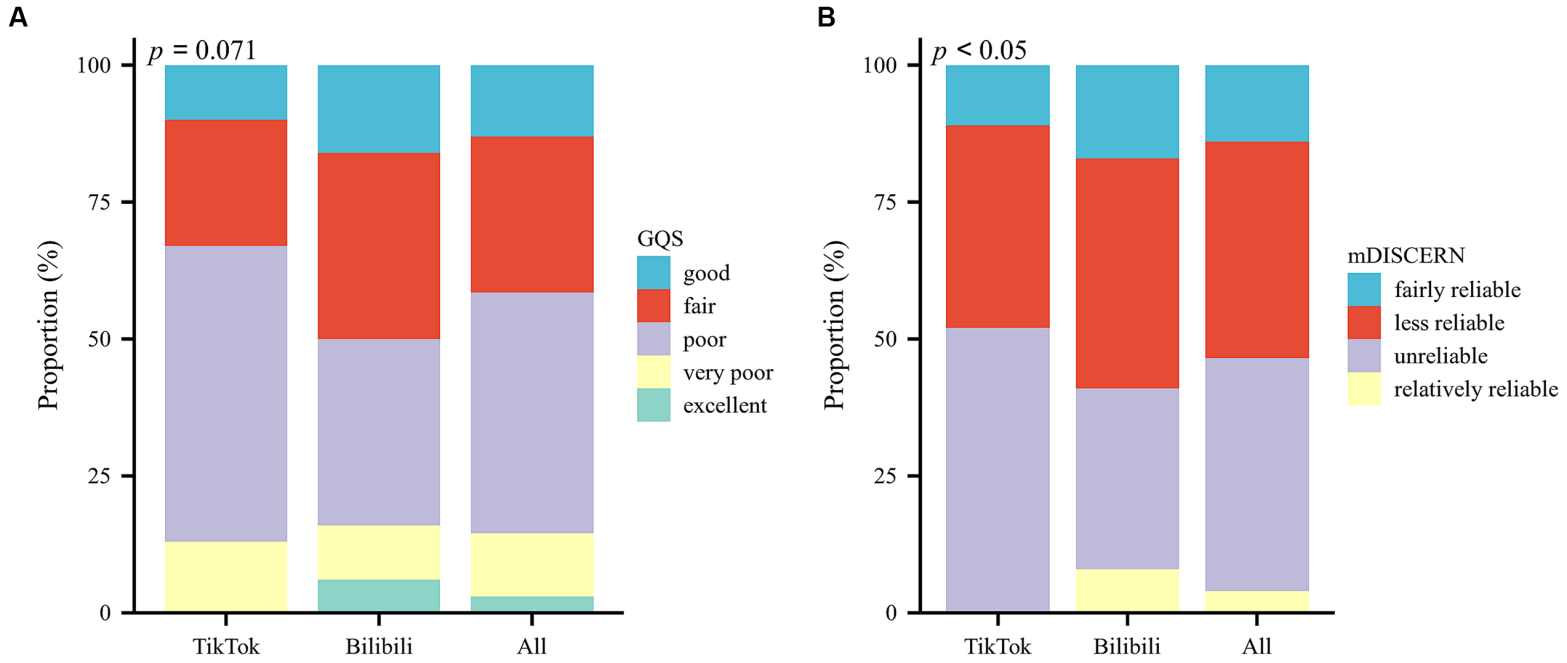
Percentage of scores on different platforms, **(A)** GQS scores, **(B)** mDISCERN scores.

The consistency of the GQS scores and mDISCERN scores between the two observers was verified, with a kappa value of 0.793 and 0.853. In TikTok videos, the median score of GQS was 2.00 and the median score of mDISCERN was 1.00. In Bilibili videos, the median scores of GQS and mDISCERN are 3.00 and 2.00, respectively (Table 2). Figure 2 shows the score distribution of GQS and mDISCERN. Although the GQS and mDISCERN scores of Bilibili videos are significantly higher than those of TikTok videos (*p* = 0.002 and *p* = 0.001, respectively), the video quality and reliability of both platforms were poor.

#### 3.2.2 Video quality and reliability under different classification

**Figure 3:**
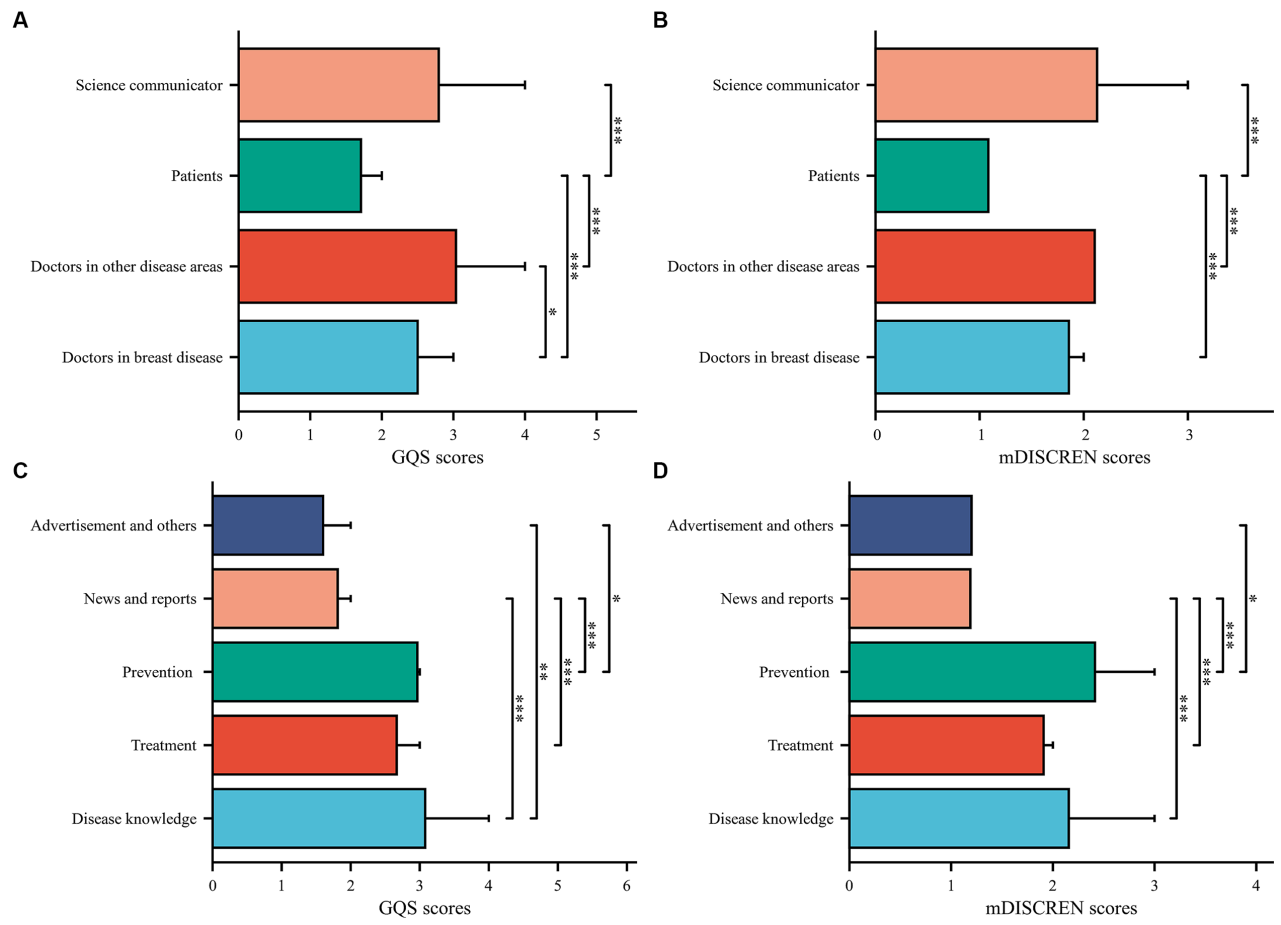
Comparison video quality and reliability of different content and different publishers. **(A,B)** Different publishers. **(C,D)** Different content; *p < 0.05; **p < 0.01; ***p < 0.001.

**Figure 4:**
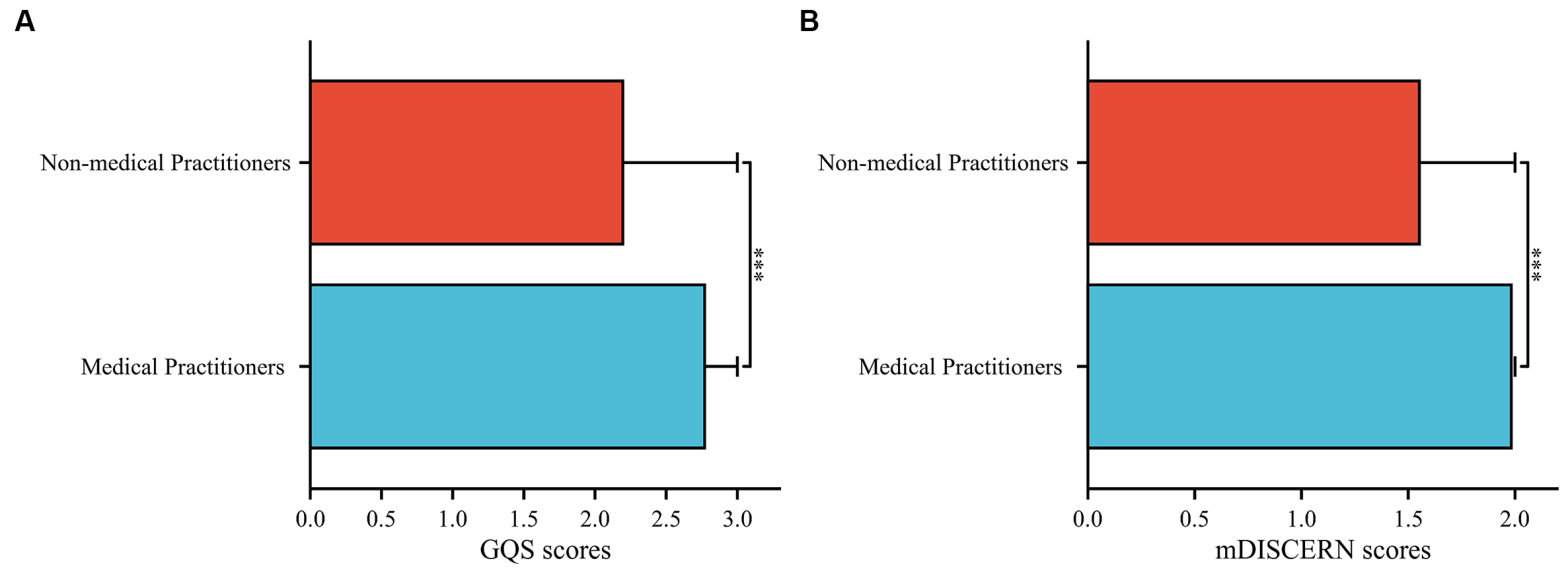
Comparison of video quality and reliability between medical practitioners and non-medical practitioners, **(A)** GQS scores, **(B)** mDISCERN scores.

**Figure 5:**
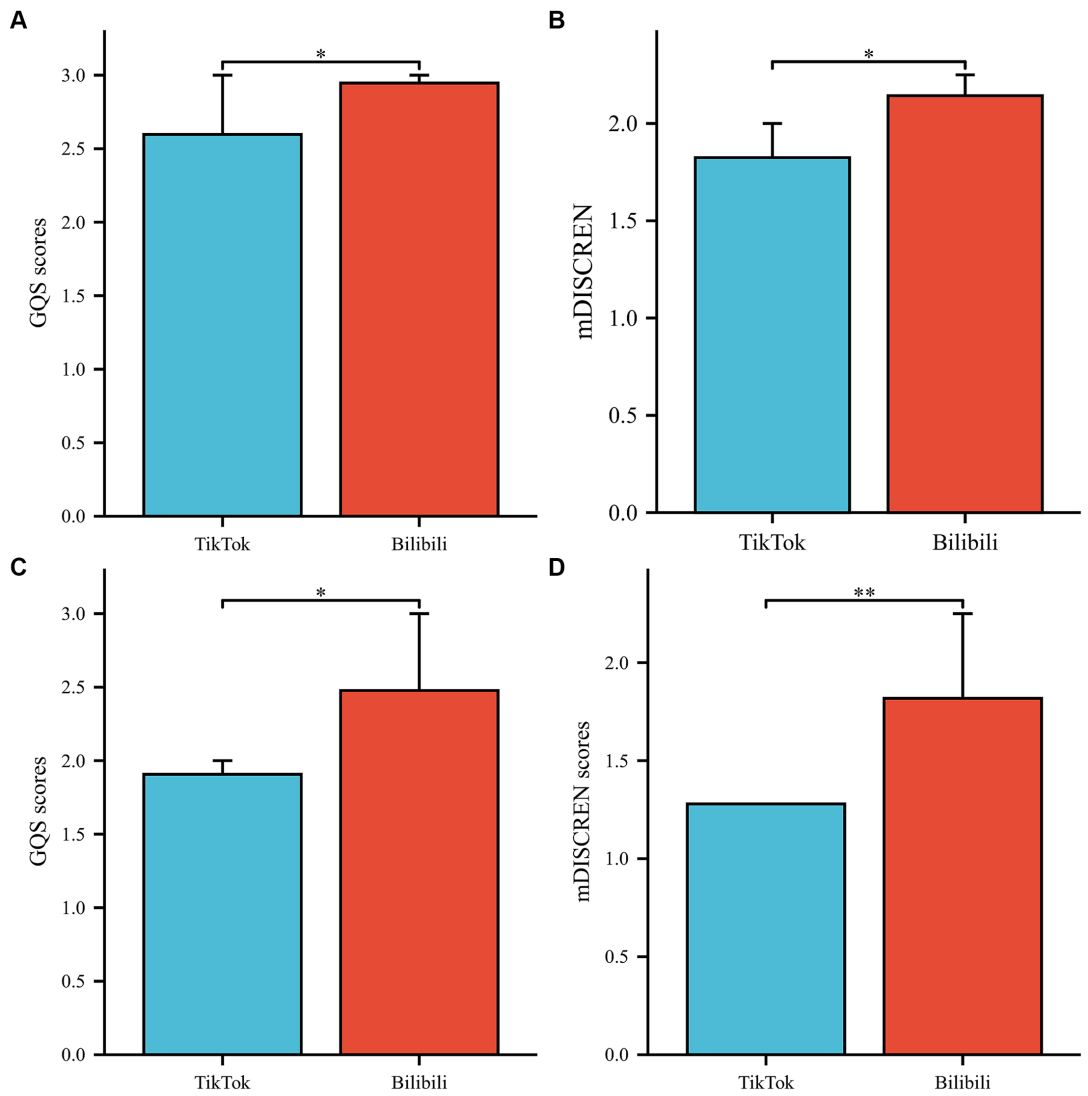
Comparison of video quality and reliability for medical practitioners and non-medical practitioners between platforms **(A,B)** Medical practitioners. **(C,D)** Non-medical practitioners; *p < 0.05, **p < 0.01.

In terms of video sources, the GQS scores of videos posted by patients was significantly lower than those posted by doctors in breast disease, doctors in other disease areas, and science communicators (all *p* < 0.001) (Figure 3A). The same was found in the mDISCERN scores of videos posted by patients (Figure 3B). It is worth noting that the GQS scores of videos posted by doctors in breast disease were significantly lower than those posted by doctors in other areas (*p* < 0.05) (Figures 3A,B). The GQS and mDISCERN scores of videos posted by medical practitioners were significantly higher than those posted by non-medical practitioners (*p* < 0.001 and *p* < 0.001, respectively) (Figure 4). Regardless of whether the videos source were medical practitioners or non-medical practitioners, the scores of GQS and mDISCERN scores of Bilibili videos were significantly higher than those of TikTok videos (all *p* < 0.001) (Figure 5).

In terms of video content, the GQS scores of news report videos were lower than those of disease knowledge, prevention and treatment videos (all *p* < 0.001). The GQS scores of videos covering disease knowledge and prevention were higher than those of advertisements videos (*p* < 0.01 and *p* < 0.05, respectively) (Figure 3C). The mDISCERN scores of news report videos were also lower than those of disease knowledge, prevention and treatment videos (all p < 0.001). The mDISCERN scores of videos containing prevention content were higher than those of advertisements videos (p < 0.05) (Figure 3D).

### 3.3 Secondary results

#### 3.3.1 Percentage of video sources and video content

There are significant statistical differences between TikTok and Bilibili in terms of video source and content (*p* < 0.001 and *p* < 0.001, respectively). Supplementary Figure S1A shows the source of the video. TikTok has the largest number of videos uploaded by doctors in breast disease, accounting for 40% (40/100), followed by patients (26/100). Bilibili has the most videos uploaded by doctors in other disease areas, accounting for 40% (40/100), and the least videos uploaded by doctors in breast disease, accounting for 16% (16/100). In terms of video content, news and reports account for the largest proportions in TikTok and Bilibili, accounting for 38% (38/100) and 31% (31/100) respectively (Supplementary Figure S1B). It is clearly shown in the radar chart (Supplementary Figure S1C) that compared with doctors in other areas, doctors in breast disease publish less disease knowledge and prevention videos, while more news and report videos; most of the videos published by patients are news and Report, that is, the patient’s own experience.

#### 3.3.2 Correlation analysis

Since the data were not normally distributed, we used Spearman correlation coefficient to analyze the relationship between different variables (Supplementary Figure S2). The following variables were positively correlated: likes and comments (*r* = 0.959, *p* < 0.001), likes and saves (*r* = 0.922, *p* < 0.001), likes and shares (*r* = 0.927, *p* < 0.001), likes and days (*r* = 0.263, *p* < 0.001), comments and saves (*r* = 0.898, *p* < 0.001), comments and shares (*r* = 0.904, *p* < 0.001), comments and days (*r* = 0.260, *p* < 0.001), saves and shares (*r* = 0.950, *p* < 0.001), duration and days (*r* = 0.195, *p* < 0.01), shares and days (*r* = 0.299, *p* < 0.001), GQS scores and mDISCERN scores (*r* = 0.767, p < 0.001), GQS scores and duration (*r* = 0.240, *p* < 0.001). The following variables were negatively correlated: GQS scores and likes (*r* = −0.191, *p* < 0.01), GQS scores and comments (*r* = −0.256, *p* < 0.001), mDISCERN scores and likes (*r* = −0.198, *p* < 0.01), mDISCERN scores and comments (*r* = −0.243, *p* < 0.001).

## 4 Discussion

### 4.1 Principal findings

In this cross-sectional study, we used GQS and mDISCERN tools to evaluate the quality and reliability of the top 100 breast cancer related videos on the TikTok and Bilibili platforms, respectively. The results showed that the quality and reliability of breast cancer related videos from TikTok and Bilibili were poor. This outcome could likely be attributed to these platforms’ lack of stringent supervision over the scientific soundness and authenticity of their video content. Of the 200 videos reviewed, only a few provided additional referential sources or mentioned areas of controversy, resulting in no videos scoring a five on the mDISCERN scale. From the point of view of video sources, the quality of videos posted by doctors in breast disease were lower than that of doctors in other areas Moreover, videos posted by patients represented the lowest quality and reliability. With respect to the video content, news reporting videos had notably lower quality and reliability than videos discussing disease knowledge, treatment, and prevention. The videos quality of TikTok were significantly lower than those of Bilibili, regardless of whether the videos source are medical practitioners (GQS, *p* < 0.05; mDISCERN, *p* < 0.05) or non-medical practitioners (GQS, p < 0.05; mDISCERN, *p* < 0.01). This may be due to that TikTok videos are shorter in duration, cover limited content, and have a higher proportion of news and reports.

### 4.2 Video source, video content and video quality

Our study found that the quality and reliability of breast cancer related videos were related to the video source and video content. In TikTok and Bilibili, more than 40% of videos were uploaded by non-medical practitioners. However, the quality and reliability of videos uploaded by non-medical practitioners were significantly lower than those of medical practitioners, which was similar to the results of previous researches (16, 25). It is worth noting that after further subgroup analysis of video sources, we found that the quality and reliability of videos uploaded by science communicators were not significantly different from those of medical practitioners, which was contrary to the findings of He et al. (16). This disparity in video quality between medical and non-medical practitioners in this study appears to emanate predominantly from patient-uploaded content. Most of the videos uploaded by patients were patient experiences. Due to the lack of medical expertise and emotional bias (26), the quality and reliability of videos uploaded by patients were significantly lower than those of the other three categories of personnel. Among medical practitioners, the quality of videos uploaded by doctors in breast disease is significantly lower than that of doctors in other areas. This may be related to a large number of outpatient videos released by doctors in breast disease. Due to the lack of systematic introduction of patients’ condition and explanation of disease knowledge, the quality of such videos is often poor. In addition, due to differences in the condition of patients, the diagnosis and treatment programs of specific patient may not be suitable for all patients, which is of little reference significance to other patients or even misleading. This suggests that doctors in breast disease should further optimize video content and send more disease-related knowledge that can benefit patients. Although TikTok and Bilibili have professionally certified medical practitioners with special identification symbols, due to the professionalism and particularity of medical videos, the platforms should formulate stricter policies to restrict patients from publishing medical videos, and strengthen the supervision of medical practitioners and science communicators to urge them to create more scientific and rigorous high-quality videos.

In the realm of video content, the quality and reliability of news videos are significantly lower than those of disease knowledge, treatment and prevention, because they were mainly consist of doctor outpatient videos and patient experience. The quality of disease knowledge, treatment and prevention videos is moderate, but the reliability is poor. In addition, we noticed that few videos cover multiple aspects, and most videos only contain one aspect of disease knowledge, treatment, and prevention. Notably, many studies have proven that palliative care can significantly improve the quality of life of patients with advanced breast cancer (27–29). Despite this, none of the videos in our study mentioned anything related to breast cancer palliative care, which is consistent with the findings of Ayoub et al. (30) on Arab breast cancer videos. This suggests that when making breast cancer-related health education videos, video uploaders should make the content more comprehensive and prevent the omission of important health information that can benefit patients.

### 4.3 Correlation between video quality and video characteristics

We found that video quality had a weak negative correlation with likes and comments, but had no obvious correlation with saves and shares. This was consistent with the results of many previous studies, suggesting that viewers have poor ability to distinguish between high-quality video and low-quality video (31, 32). On the one hand, this phenomenon occurs because medical videos with health education significance are highly professional, which is difficult for ordinary viewers with limited knowledge to understand, so it is difficult to become interested in them; while patient experience videos are closer to life, viewers are more willing to like and comment. On the other hand, due to the push mechanism of TikTok’s Bilibili, videos with higher likes may be ranked higher and gain more exposure, which aggravates this phenomenon.

In addition, we found that there is a significant positive correlation between video duration and video quality, because long videos can accommodate more information. Although there is no significant correlation between video length and video popularity (likes, comments, saves, shares) in our study, in other studies, viewers prefer health education videos with shorter duration (30, 33). Therefore, how to balance the video duration and content richness, that is, to deliver higher-quality medical and health videos to the audience in the shortest possible time and in easier-to-understand language, is worthy of consideration by video publishers.

### 4.4 Evaluation of quantitative scoring tools

Different from the two previous single-platform breast cancer video quality assessment studies, our study used the mDISCERN tool instead of the DISCERN tool to assess video reliability (34, 35). Previous studies have pointed out that the DISCERN tool is not suitable for evaluating video materials because it was developed to enable patients and informants to judge the quality of written information (19, 36). The mDISCERN tool was adapted from the DISCERN tool, which is a 5-point scale that is more suitable for assessing the reliability of videos (23). For the quality of video, we use the GQS scale to evaluate. GQS was originally developed by Bernard et al. to evaluate the quality of information on websites, and was later widely used to assess the quality of health education videos, and its effectiveness has been confirmed in previous studies (20, 37, 38). However, these two tools have certain limitations. They only focus on the textual content of the video and do not evaluate the visual effects and comment content of the video, which is an aspect that needs to be improved in future video evaluation tools.

### 4.5 Practical significance

The disease burden of breast cancer is increasing in China (2). This burden can be reduced by increasing awareness of breast cancer, promoting healthy lifestyles and breastfeeding (2). Early detection and timely treatment of breast cancer can improve the prognosis of breast cancer (39, 40). The increased accessibility of the Internet has made it a primary source of health information for people. However, due to the lack of effective regulatory mechanisms, the quality of information on the Internet, especially health education videos, is often uneven, and patients are often unable to judge the quality and reliability of the videos (20, 41, 42). High-quality health education videos can improve people’s awareness of breast cancer and play a good health education role. In the study by Galiano-Castllo et al. (43), an Internet-based exercise intervention improved the quality of life of breast cancer patients. However, some misleading videos may cause people to make wrong health decisions (44, 45). Therefore, it is important to evaluate breast cancer-related videos using scientific scoring tools, which can provide suggestions and directions for video social platforms and video uploaders on how to better disseminate reliable information to health seekers. We recommend that video social platforms establish a unique section for medical videos, set up a health information review team to evaluate the content of the videos, do not allow false or incorrect videos to be published, and modify the ranking mechanism of medical education videos to ensure videos with higher quality and reliability are ranked higher. For medical practitioners, they should establish a rigorous attitude and release more comprehensive and high-quality videos to better guide patients.

### 4.6 Advantages and limitations

As far as we know, this is the first study on the quality of breast cancer videos from two major video platforms in China from the perspective of breast surgeons. In terms of evaluation tools, we used the mDISCERN tool, which is more suitable for evaluating video materials; and used both the GQS and mDISCERN tools to comprehensively and systematically evaluate the quality and reliability of the videos. In addition, the video is graded by two raters, and any differences are fully discussed with the arbitrator and then decided by the arbitrator, which greatly reduces the differences in video scoring caused by individual subjective factors.

However, our study also has limitations. First of all, we only collected the top 100 videos on the two platforms, not all videos, but existing research have proven that some of the top 100 videos as a representative video set can reflect the overall quality of video in this field (17, 32, 46). Second, this is a cross-sectional study, with the passage of time, the content of the video platform will change. Third, our study focused on Chinese videos on Chinese video social platforms and lacked analysis and comparison of English videos, so the research results may not be generalizable to video platforms in other countries. However, breast cancer is a disease of global concern, and more cross-national and cross-language studies are needed to fill this gap in the future.

## 5 Conclusion

The rising incidence and mortality of breast cancer has put considerable pressure on China’s medical and economic sectors. However, people’s understanding of breast cancer is limited. Video social platforms, currently the main medium for disseminating health information, provide health seekers with access to information on the prevention, diagnosis, and treatment of their diseases. However, our study results show that the quality and reliability of breast cancer videos on TikTok and Bilibili are poor, and the overall quality is unsatisfactory. Generally speaking, the quality of videos uploaded by medical practitioners on disease knowledge, prevention and treatment is high, while the quality of videos posted by patients is extremely low and cannot serve as a good health education. Video social platforms should formulate relevant policies to supervise and review medical videos. We encourage medical practitioners to publish more accurate and higher-quality videos as a reliable source of health information for the public.

## Data availability statement

The original contributions presented in the study are included in the article/Supplementary material, further inquiries can be directed to the corresponding authors.

## Author contributions

HuL: Conceptualization, Data curation, Investigation, Software, Visualization, Writing – original draft. JP: Conceptualization, Data curation, Investigation, Methodology, Software, Writing – original draft. LL: Conceptualization, Formal analysis, Investigation, Methodology, Visualization, Writing – review & editing. AD: Investigation, Methodology, Supervision, Writing – review & editing. XH: Formal analysis, Investigation, Software, Supervision, Visualization, Writing – review & editing. GY: Formal analysis, Methodology, Project administration, Validation, Writing – review & editing. JM: Formal analysis, Investigation, Resources, Writing – review & editing. HaL: Data curation, Funding acquisition, Project administration, Supervision, Validation, Writing – review & editing. YL: Conceptualization, Data curation, Funding acquisition, Project administration, Supervision, Validation, Visualization, Writing – review & editing.
