# Supplementary material for: Assessment of the reliability and quality of breast cancer related videos on TikTok and Bilibili: cross-sectional study in China
Source: Front Public Health. 2024 Jan 22;11:1296386. doi: 10.3389/fpubh.2023.1296386 (PMC10839971; doi:10.3389/fpubh.2023.1296386)
Supplement: Supplementary file 1 [file Data_Sheet_1.docx]

***Supplementary Material***

**Supplementary table 1.** Global Quality Score (GQS) scoring standard. (Scoring ranges from 1 to 5)

| **GQS description** | **Score** |
| --- | --- |
| Poor quality, poor flow of the site, most information missing, not at all useful for patients | 1 |
| Generally poor quality and poor flow, some information listed but many important topics missing, of very limited use to patients | 2 |
| Moderate quality, suboptimal flow, some important information is adequately discussed but others poorly discussed, somewhat useful for patients | 3 |
| Good quality and generally good flow, most of the relevant information is listed, but some topics not covered, useful for patients | 4 |
| Excellent quality and excellent flow, very useful for patients | 5 |

**Supplementary table 2.** modified DISCERN (mDISCERN) scoring standard. (1 point for answer ‘yes’, 0 point for answer ‘no’)

| **Reliability Score** |
| --- |
| 1. Is the video clear, concise, and understandable? |
| 2. Are valid sources cited? |
| 3. Is the content presented balanced and unbiased? |
| 4. Are additional sources of content listed for patient reference? |
| 5. Are areas of uncertainty mentioned? |

**Supplementary table 3.** Score grading for Global quality score and modified DISCERN tools.

| Scale, score | Level |
| --- | --- |
| Global quality score |  |
| 1 | Very poor |
| 2 | Poor |
| 3 | Fair |
| 4 | Good |
| 5 | Excellent |
| modified DISCERN |  |
| 1 | Unreliable |
| 2 | Less reliable |
| 3 | Generally reliable |
| 4 | Reliable |
| 5 | Very reliable |

**
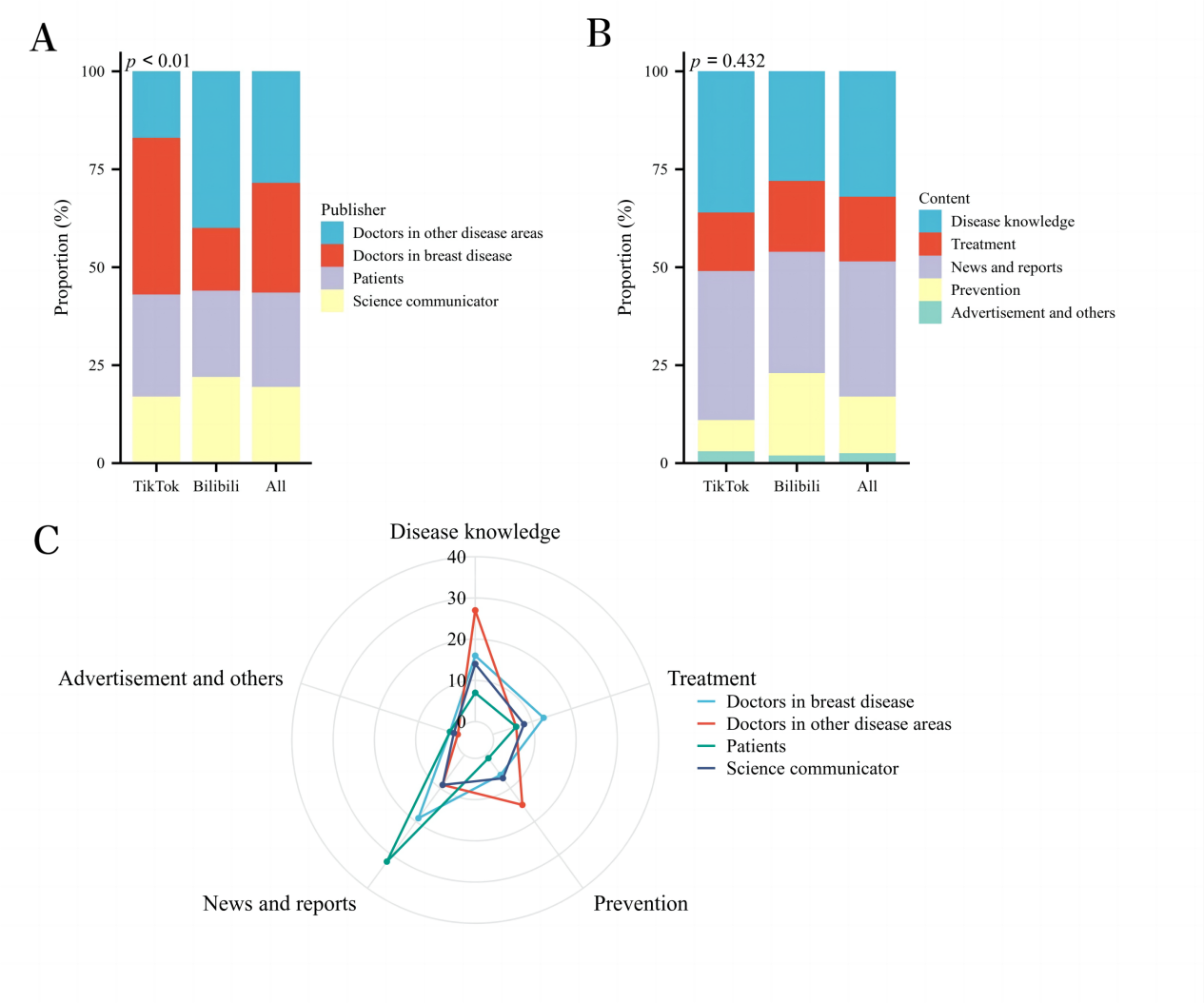
Supplementary figure 1.** Percentage of different content and different publishers across the three platforms and overall. ((A) Different content. (B) Different publishers. (C) Different content in different publishers.)


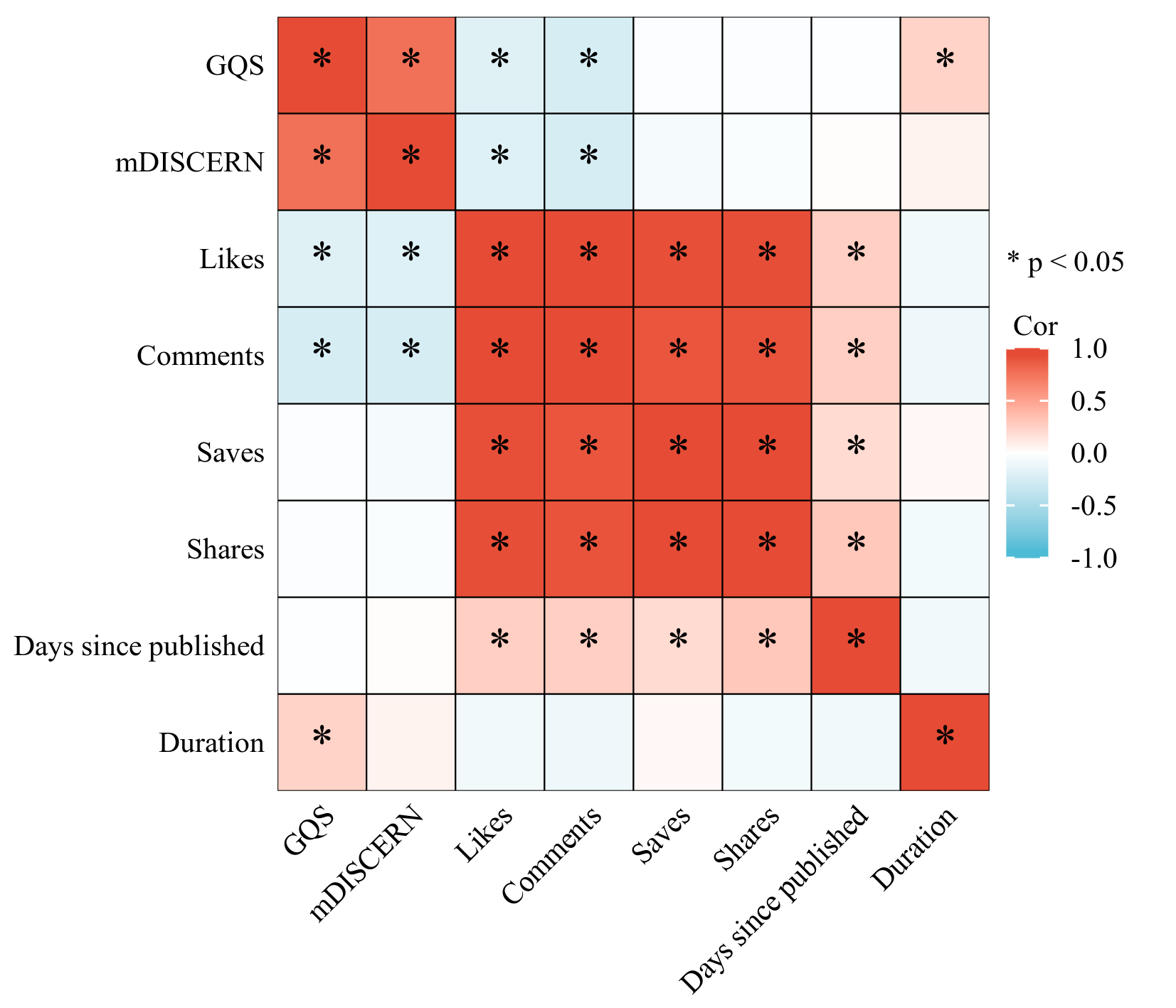


**Supplementary figure 2.** Correlation analysis between different parameters.
